# Comparative effectiveness of open versus laparoscopic resection in patients with locally advanced colon cancer: Inverse probability of treatment weighting approach

**DOI:** 10.1371/journal.pone.0338741

**Published:** 2025-12-29

**Authors:** Mesnad S. Alyabsi, Anwar H. Alqarni, Adel F. Almutairi, Mohammed S. Alhalafi, Mohammed A. Algarni, Kanan M. Alshammari, Nahar A. Alselaim

**Affiliations:** 1 Population Health Research Section, King Abdullah International Medical Research Center, Riyadh, Saudi Arabia; 2 King Saud bin Abdulaziz University for Health Sciences, Riyadh, Saudi Arabia; 3 King Abdullah International Medical Research Center, Riyadh, Saudi Arabia; 4 Surgery Department, Ministry of National Guard - Health Affairs, Riyadh, Saudi Arabia; 5 Oncology Department, Ministry of National Guard - Health Affairs, Riyadh, Saudi Arabia; Athens Medical Group, Psychiko Clinic, GREECE

## Abstract

**Background:**

In Saudi Arabia, colorectal cancer is one of the most common cancers. Most of colorectal cancer cases were diagnosed at an advanced stage, making the treatment process more challenging. In this paper, we aim to investigate the differences between open and laparoscopic colectomies and factors influencing the use of these approaches in locally advanced colon cancer patients.

**Methods:**

The study population included patients with locally advanced colon cancer at the National Guard-Health Affairs, Saudi Arabia. Descriptive and clinical data were collected for the patients diagnosed with locally advanced colon cancer from 2016 to 2021. The variables related to the locally advanced colon cancer patients’ characteristics were compared with a chi-square test, Fisher’s exact tests, student’s t-tests, or Wilcoxon tests when appropriate. The overall survival and recurrence-free survival were computed using the Cox regression model.

**Results:**

Of the 222 patients who underwent tumor resection, 75 (33.8%) underwent a laparoscopic colectomy. Unlike open colectomy patients, the group with laparoscopic colectomy were younger, had a higher body mass index, and had tumors in the left colon, with ≥12 harvested lymph nodes. The weighted data indicated no difference in the 5-year overall-survival, and the recurrence-free survival between the approaches [5-year overall survival, hazard ratio: 0.42, 95%CI (0.16, 1.12); recurrence-free survival, hazard ratio: 0.74, 95%CI (0.31, 1.78)].

**Conclusions:**

No significant differences were observed between laparoscopic and open surgical approach for patients with locally advanced colon cancer.

## Introduction

Colorectal cancer (CRC) is a common malignancy with more than 1.9 million incident cases and 904,000 deaths reported globally in 2022 [1]. Prediction studies estimate that the number of cases will increase to 3.2 million by the year 2040 [2]. In 2024, approximately 152,729 cases of CRC cancer are expected to be diagnosed in the United States, resulting in an estimated 53,010 deaths. [3] Notably, over 50% of CRC cases are diagnosed at a local or regional stage [4,5]. Locally advanced colon cancer (LACC) is a subtype that includes stage IIb/c and stage III tumors. Stage IIb/c cancers have grown into the colon’s outermost layer, and stage III tumors have spread to nearby lymph nodes [6,7]. LACC is a serious condition with a survival rate varying between 25% and 60% depending on several factors such as the depth of invasion (T stage) and the number of involved lymph nodes (N stage) [8]. Such disparate survival rates underscore the importance of timely and effective treatment, which may include the tumor resection approach (laparoscopic or open), chemotherapy, or a combination of these treatments.

Though the National Comprehensive Cancer Network (NCCN) recommends a surgical resection for LACC, the optimal surgical approach is contentious [9]. Laparoscopic colectomy (LC), emerging as a minimally invasive substitute for open colectomy (OC), has gained traction in recent decades. Previous studies indicate the advantages of LC in decreasing short-term complications such as shorter hospitalization, less postoperative pain, and lower risk of complications during surgery [10,11]. According to the American Society of Colon and Rectal Surgeons (ASCRS), laparoscopic excision of locally advanced tumors offers a safe and effective treatment with comparable long-term outcomes to OC [12]. However, the NCCN guidelines do not recommend laparoscopic resection for T4 tumors [9]. Unlike OC, LC poses challenges in achieving microscopic negative margins (R0) and performing multivisceral resection, especially in advanced tumors [13]. Though some studies reported technical complexities and worse perioperative outcomes for the LC approach, others advocate for its superiority, even in obese patients. [14–16].

CRC is a commonly diagnosed cancer in Saudi Arabia, particularly in males, in whom it is the most diagnosed cancer. For females, it ranks third after breast and thyroid cancers [17]. More than two-thirds of CRC patients are diagnosed with advanced disease. The age-adjusted incidence rates per 10^5^ people were 13.9 for males and 11.3 for females in the latest report from 2018 [18]. In recent years, the incidence rates of early- and late-onset CRC have shown an upward trends, with constant increase in the average annual percentage change among early-onset patients [19]. The Clinical Outcomes of Surgical Therapy (COST) Study Group trial, a pivotal randomized controlled trial, excluded patients with T4 tumors and transverse colon cancers. As a result, the evidence base from this trial may not be generalizable to patients with locally advanced colon cancer (LACC), underscoring the need for focused research in this subgroup [11]. The present study characterizes patients with LACC who underwent OC versus LC and identifies factors associated with the use of each approach using the inverse probability of treatment weighting (IPTW). In addition, compare the survival outcomes between these two approaches.

## Patients and methods

### Data sources

We conducted a retrospective cohort study using the electronic medical record system of the National Guard-Health Affairs (NGHA) linked with the cancer registry. The dataset included all individuals with colon cancer who received treatment at the NGHA in Riyadh. The need for informed consent has been waived as all patients at the hospital provide consent at the outset of their visits, authorizing the use of their data for research purposes while ensuring the privacy and anonymity of all individuals involved. Approval for the study was obtained from the King Abdullah International Medical Research Center Institutional Review Board, with project number NRC21R/458/11. All extracted data were de-identified, and all methods were performed in accordance with the Declaration of Helsinki. The first access to the patients’ data was on May 20, 2023. The datasets generated or analyzed during the present study are not publicly available due to patients’ privacy but are available from the corresponding author on reasonable request.

### Study population

The study included individuals with non-metastatic disease (M0) with tumor invasion beyond the muscular layer of the colon. This included patients without lymph node involvement (N0) who had tumors extending into the surrounding fat (T3), penetrating the outer lining of the colon (T4a), or invading nearby organs (T4b), corresponding to AJCC stages IIA to IIC. Additionally, patients with regional lymph node involvement (N1–N2), classified as Stage III, were also considered locally advanced patients. We restricted our population to patients aged 18 years or older, with a minimum of 6 months enrollment in the NGHA before cancer diagnosis. An additional criterion was tumor resection surgery without prior chemotherapy sessions during the study period from 2016–2021.

### Study variables

#### Patient and tumor characteristics.

The demographic and clinical characteristics extracted from the hospital records encompassed age at diagnosis, gender, body mass index (BMI), Charlson Comorbidity Index (CCI), smoking status and detailed tumor information including stage, location, number of harvested lymph nodes, and chemotherapy status. The tumor stage was identified at the time of diagnosis using the TNM staging system, (T) was categorized as T1 or T2, T3, and T4, based on the extent of malignant cell invasion to the nearby tissues. The value of N indicated lymph node involvement. N0 denote lack of involvement, N1 1–3 lymph nodes involved, and N2 4 or more metastatic regional lymph nodes. There were no missing values except for the TNM staging. As < 3% of the TNM stages were missing, we reported them in a separate category as missing variables. Tumor location was classified using the ICD for Oncology-third edition topography as follows: right colon (i.e., cecum, ascending colon, hepatic flexure of colon and transverse colon), left colon (i.e., splenic flexure of colon, descending colon, and sigmoid). The length of hospital stay, operation time, time between surgery and chemotherapy administration, tumor recurrence site, and survival outcomes were also calculated. Complications were categorized as follows: surgical site infections (SSI), gastrointestinal complications included anastomotic leaks, bowel obstruction, and abdominal wall collections, genitourinary complications included urinary tract infections, pyelonephritis, and acute kidney injury related to urinary events, circulatory complications included deep vein thrombosis, mesenteric vein thrombosis, pulmonary embolism, and myocardial infarction, pulmonary complications pulmonary complications included pneumonia, respiratory failure, and pleural effusions, and neurology.

#### Variable selection.

A propensity score based model was built to analyze the impact of the surgical approaches on patient mortality by improving the comparability of the two groups, ensuring that the standardized mean difference between them is less than 10%. Similar to previous research [20], the a priori model comprised of variables believed to confound the approach-survival association, and was chosen based on prior research. The priori selected variables were gender, age, BMI, CCI, smoking, adjuvant chemotherapy, tumor location, and the number of harvested lymph nodes (<12 vs. ≥ 12).

### Outcome variables

The outcomes of this study were the differences in the 5-year overall survival and recurrence-free survival between the patients who underwent OC vs. LC.

### Statistical analysis

The descriptive data are presented as frequency and for the categorical variables, and the mean or median were used for the numerical variables. Baseline covariate balance was assessed using the standardized mean difference (SMD) with no hypothesis testing was performed. Comparative analyses were performed using Chi-square tests, Fisher’s exact tests, two-tailed student’s t-tests, or Wilcoxon tests as applicable. In addition, the Cox proportional hazards models were built to estimate the 5-year overall survival and recurrence-free survival. Outliers were assessed using a sensitivity analysis to assess their effects on the outcomes. Overall, a *P-value*<0.05 was considered statistically significant, and due to the multiple comparisons, we have followed the Benjamini-Hochberg procedure to correct for complications’ and recurrent sites’ *p-values*. Statistical analysis was performed using SAS statistical software version 9.4 (SAS Institute Inc. Cary, NC).

#### Propensity score.

The IPTW method was used to examine the differences in the effectiveness of OC and LC. IPTW creates a pseudo-population with balanced measured covariates, thus adjusting for both confounding and selection bias [21]. Two types of weights were estimated, treatment weights (LC vs OC) and censoring weights. The treatment weights were estimated through the propensity score using logistic regression models, with the resulting weights stabilized (by multiplying weights by the marginal probability of surgical approach in the group who underwent LC and one minus marginal probability in patients who underwent OC) and truncated (at the 1–99% percentiles).

Censoring weights were computed using a pooled logistic regression model for each patient visit during the three years. To estimate the numerator of the censoring weights, the drop-out from the study was the outcome and surgery approach and time as covariates of the pooled logistic regression. For the denominator, we used the dropout as an outcome and the covariates used in treatment initiation above plus the surgical approach and time as covariates. The weights estimated from the dropout model were multiplied by those estimated from the treatment model to compute the final weights.

The final weights were used in the Cox regression model with the surgical approach as the covariate and death as an outcome, weighted by the final estimated weights (both age and BMI were entered into the original model as restricted quadratic splines with 4 knots.). We followed the patients from the date of surgery until death or censoring. Given the IPTW methodology used in this study, 95% CIs were estimated using a robust standard error.

## Results

**Fig 1:**
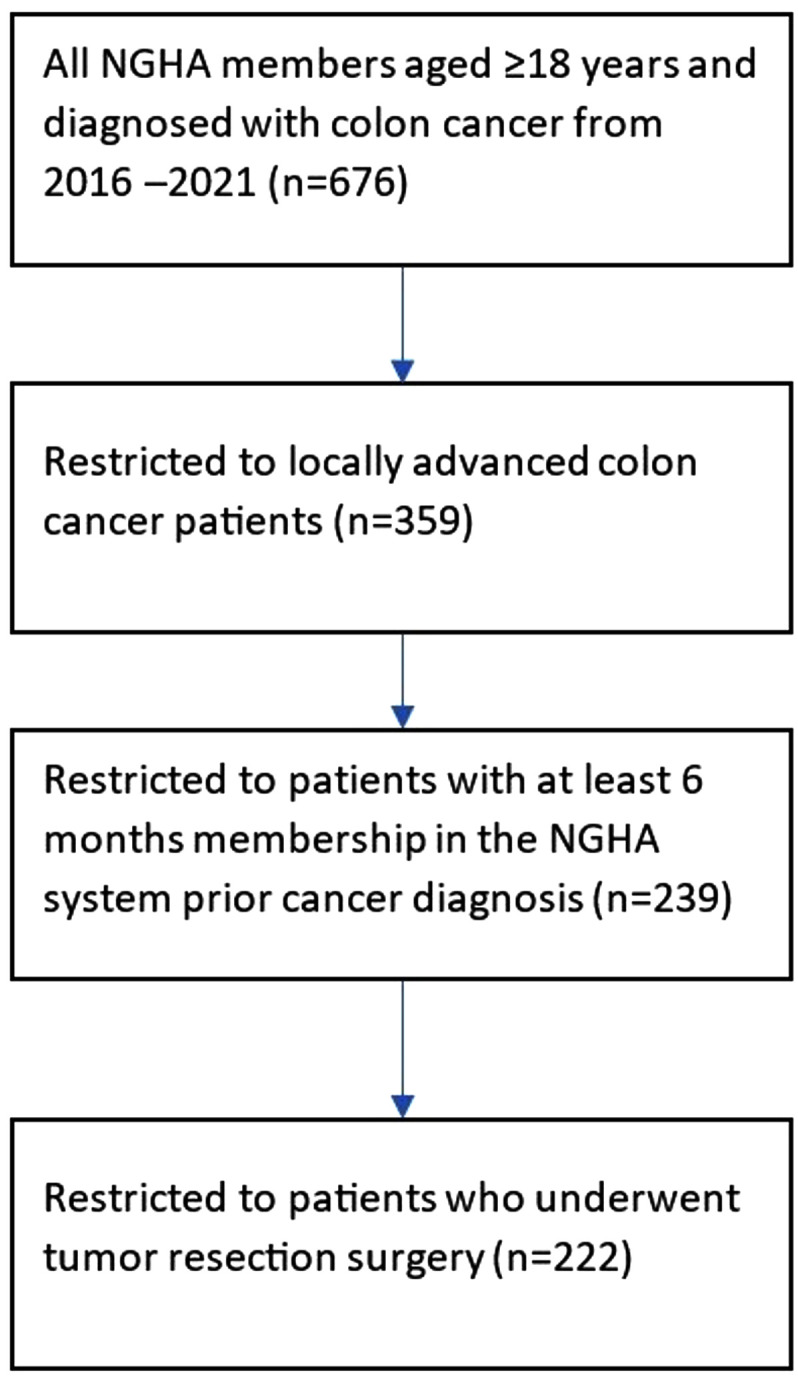
Eligibility criteria of the study population.

**Fig 2:**
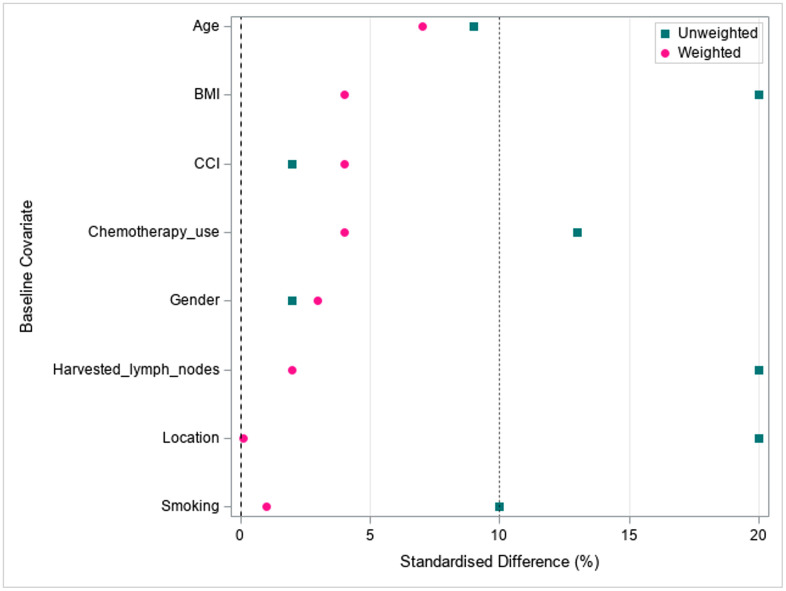
Standardized mean difference (SMD) balance plot before and after propensity score matching.

Of the 676 patients eligible for this study, we restricted our study population to the group with LACC who underwent surgical resection and were members of the NGHA for at least 6-months before the cancer diagnosis (**Fig 1**). The median follow-up time was 30.85 (26.07) months. During the study period, 222 LACC patients underwent tumor resection surgery, of which 147 (66.2%) underwent the OC approach (**Table 1**). In contrast to the patients who underwent OC, the group with LC were younger (60.97 vs. 62.32 years), had a numerically higher BMI, were diagnosed at stage III, presented with tumors located in the left colon, and had a higher proportion of patients with ≥12 harvested lymph nodes. Conversely, the OC patients had more comorbidities, were mostly non-smokers, and had received chemotherapy. After the propensity score adjustment, the mean age, BMI value, and smoking status of patients was similar in both treatment groups (age: 61.72 vs. 60.79, BMI: 28.53 vs. 28.33, smoking: 90.0% vs. 90.2%). The balance plot demonstrated that all covariates achieved adequate balance after propensity score adjustment, with standardized mean differences reduced to below the 10% (**Fig 2**).

**Table 1:** Baseline characteristics of unweighted and weighted data.

	Unweighted			IPTW		
	Open (n = 147)	Lap (n = 75)	SMD	Open (n = 146.8)	Lap (n = 74.2)	SMD
**Age**			0.09			0.07
Mean (SD)	62.32 (13.54)	60.97 (13.47)		61.72 (13.40)	60.79 (13.47)	
**Gender**			0.02			0.03
Male	90 (61.2)	46 (61.3)		84.54 (60.6)	43.90 (59.2)	
**BMI**			0.20			0.04
Mean (SD)	28.25 (6.1)	29.50 (5.8)		28.53 (5.9)	28.33 (5.8)	
**Smoking**			0.10			0.01
Non-smoker	134 (93.0)	65 (90.2)		125.36 (90.0)	66.85 (90.2)	
**CCI**			0.02			0.04
0	28 (19.0)	15 (20.0)		26.80 (19.2)	12.41 (16.7)	
1	19 (12.9)	10 (13.3)		16.48 (11.8)	9.88 (13.3)	
≥ 2	100 (68.0)	50 (66.6)		90.0 (68.9)	51.79 (69.9)	
**Tumor stage**			0.78			
II	68 (46.2)	34 (45.3)		68 (46.2)	34 (45.3)	
III	75 (51.0)	40 (53.3)		75 (51.0)	40 (53.3)	
Missing	4 (2.7)	1 (1.3)		4 (2.7)	1 (1.3)	
**TNM (T)**						
T1 or T2	6 (4.0)	8 (10.6)	0.03	6 (4.0)	8 (10.6)	
T3	94 (63.5)	55 (73.3)		94 (63.5)	55 (73.3)	
T4	43 (29.5)	11 (14.6)		43 (29.5)	11 (14.6)	
Missing	4 (2.7)	1 (1.3)		4 (2.7)	1 (1.3)	
**TNM (N)**			0.89			
N0	68 (46.2)	34 (45.3)		68 (46.2)	34 (45.3)	
N1	53 (36.0)	27 (36.0)		53 (36.0)	27 (36.0)	
N2 or N3	22 (14.9)	13 (17.3)		22 (14.9)	13 (17.3)	
Missing	4 (2.7)	1 (1.3)		4 (2.7)	1 (1.3)	
**Chemotherapy use**			0.13			0.04
Yes	94 (63.9)	43 (57.3)		88.95 (63.8)	48.29 (65.2)	
**Location**			0.20			**0.001**
Right colon	64 (43.5)	26 (34.6)		58.42 (41.9)	30.75 (41.5)	
Left colon	83 (56.4)	49 (65.3)		80.86 (58.0)	43.31 (58.4)	
**Harvested lymph nodes**			0.20			0.02
< 12	17 (11.5)	5 (6.6)		14.67 (10.5)	7.43 (10.0)	
≥ 12	123 (83.6)	69 (92.0)		124.61 (89.4)	66.65 (89.9)	

As shown in **Table 2**, both unweighted and weighted data showed no differences in 5-year overall survival, and the recurrence-free survival between the two surgical approaches [5-year OS, HR: 0.42, 95%CI (0.16, 1.12); RFS, HR: 0.74, 95%CI (0.31, 1.78)].

**Table 2:** Multivariate analysis of the 5-year overall and recurrence-free survival comparing laparoscopic with open surgery for LACC.

	Unweighted data			Weighted data		
	HR	95% CI	P	HR	95% CI	P
**OS**	0.63	(0.23,1.73)	.37	0.42	(0.16,1.12)	.08
**RFS**	0.65	(0.27,1.55)	.33	0.74	(0.31,1.78)	.50

OS: Overall survival; RFS: Recurrence-free survival; HR: Hazard ratio; IPTW: Inverse probability of treatment weighting.

**Table 3** illustrates the short-term and long-term outcomes of the surgery. The OC approach resulted in a higher percentage of anastomotic leak (5.4%), increased hemorrhage necessitating blood transfusion (23.1%), and longer hospital stay postoperatively (18.40 vs. 9.71 days, *P-value*= < 0.001). However, the OC operations had a shorter operation time than the LC (212.22 vs. 248.72 min, *P-value*= < 0.001). A slightly higher percentage of LC patients received chemotherapy within the first 30 days following their surgery (22.7% vs. 19.7%), though the difference was not significant.

**Table 3:** Other outcomes.

Patients’ outcomes	Open (n = 147)	Lap (n = 75)	p-value
Mean length of operation in minutes (SD)	212.22 (81.55)	248.72 (84.03)	**<0.001**
Mean length of hospital stays in days (SD)	18.40 (28.56)	9.71 (5.23)	**<0.001**
Anastomotic leak	8 (5.4)	1 (1.3)	0.14
Bleeding requiring blood transfusion	34 (23.1)	10 (13.3)	0.08
Chemotherapy within 30 days after surgery	29 (19.7)	17 (22.6)	0.39

Lastly, as depicted in **Table 4**, surgical site infection appeared as the most prevalent postoperative complication in both groups (23.8% in OC vs. 9.3% in LC). The liver was considered the most recurrent site in the OC group (6.8%), while the peritoneum had the highest percentage of recurrent tumor sites in the LC group (4.0%) as well as other different locations. However, none of the p-values for postoperative complications or recurrence sites remain statistically significant after performing the Benjamini-Hochberg procedure to correct for the multiple comparisons.

**Table 4:** Surgical complications and most recurrent site.

	Open (n = 147)	Lap (n = 75)	p-value	Adjusted p-value
SSI	35 (23.8)	7 (9.3)	0.01	0.06
Circulatory	12 (8.1)	1 (1.3)	0.09	0.14
Pulmonary	12 (8.1)	1 (1.3)	0.09	0.14
GI	17 (11.5)	1 (1.3)	0.02	0.06
GU	7 (4.7)	3 (4.0)	0.74	0.74
Neurology	1 (0.6)	0 (0.0)	0.59	0.71
Common recurrent site	**Open (n = 147)**	**Lap (n = 75)**	**p-value**	**Adjusted p-value**
Liver	10 (6.8)	1 (1.3)	0.09	0.20
Peritoneum	7 (4.7)	3 (4.0)	0.16	0.20
Anastomotic site	4 (2.7)	1 (1.3)	0.22	0.22
Lung	3 (2.0)	0 (0.0)	0.16	0.20
Others	5 (3.4)	3 (4.0)	0.10	0.20

SSI: Surgical Site Infection; GI: Gastrointestinal; GU: Genitourinary

## Discussion

The motivation for designing this study was the uncertainty surrounding the incompleteness of tumor resection using LC approach and the absence of large RCTs comparing LC versus OC approach in LACC patients. Prior RCTs excluded LACC patients from their analyses [11]. Given that incomplete resection is negatively affecting the survival of LACC patients, the current study attempted to answer a timely question regarding the survival of LACC patients according to surgical approach. Using an IPTW method, we investigated the 5-year overall survival and recurrence-free survival between the LC and OC approaches and found that, although LC showed a better outcomes compared to OC, the multivariate analysis appeared to be statistically insignificant.

Though the ASCRS equates the long-term oncologic outcomes of LC and OC approaches, both the NCCN and the European Association of Endoscopic Surgery (EAES) discourage LC for LACC patients. These discrepancies in recommendations stem from inadequate evidence regarding the suitable surgical approach for LACC patients. This study strived to add to the existing literature of observational studies in LACC.

Several prior observational studies compared the effectiveness of LC versus OC [22–24]. In a German study that assessed and compared the oncologic outcomes between LC and OC colon cancer patients, the authors found no significant overall survival difference between the compared groups [HR: 0.81; 95% CI (0.61,1.06)] [23]. Our IPTW analyses align with these results, showing no dissimilarity in the 5-year overall survival. Notably, the German study found a significant difference favoring the LC approach in colon cancer patients with T1-3N0 tumors, a subgroup we did not investigate, since we limited our patients to advanced stage II and stage III. Similarly, a Korean study, focusing mainly on T4 tumors, found no differences in the 3-year survival between the compared groups, but the authors only reported the overall survival without providing the adjusted results using regression methods [24]. With regard to the recurrence-free survival, there was an insignificant difference between the OC and LC patients.

The current study found no differences in the effectiveness for the various subpopulations. For instance, the LC approach had the same effectiveness in obese or overweight patients, which is similar to some studies [22,25,26] but not others [14,15]. Further, the LC patients experienced shorter hospital stays than the OC patients (9 vs.18 days), a significant finding given its implications for the timely initiation of ACT, vital for expedited recovery and effective treatment [27,28]. Given the similar 5-year survival and recurrence-free survival between the two approaches and the faster recovery in the LC patients, it could be a valuable option for patients prone to delayed recovery that might compromise their ACT initiation.

In our study, LC was more frequently performed in younger patients. This trend may reflect the demographic characteristics of the Saudi population, which has a relatively youthful population compared to developed countries. While the LC approach is minimally invasive procedure, clinical considerations, including comorbidities and performance status, may contraindicate its use in older patients.

Our results showed that the number of surgical complications were fewer in the LC group, a reflection of the smaller incisions compared to OC. The larger OC incisions have high complication risks, such as increased blood loss, postoperative pain, longer hospital stay, and wound infections [29–32]. This concurs with literature showing significantly lower complications in LC patients [29,33]. Though surgical site infections are a potential risk in both approaches, they were most common in the OC patients (56.7%), as reported [30]. Though not statistically significant, the LC group had a higher peritoneum recurrence than the OC (37.5% vs 24.1%), similar to a study investigating the long term outcomes of LACC and another retrospective study focusing on T4 colon cancer patients [34] but not others [35].

Our experience showed similarities and distinctions when compared to the current standards of care in developed countries such as Europe and the USA. LC has become widespread approach for LACC due to the availability of advanced surgical technologies, and robust training programs. On the other hand, in Saudi Arabia, factors including healthcare infrastructure, access to advanced surgical equipment, and trained surgeons influence surgical decisions. These differences highlight the importance of adapting international best practices to fit local healthcare settings.

Though we endeavored to minimize residual confounding and selection bias, it remains possible that the surgeon’s choice of the type of surgical approach used is based on their sole judgment which was not captured in the data. Additionally, the findings of the current study are derived from the NGHA population, a healthcare system with experienced surgeons, which might not reflect the practice in other systems in Saudi Arabia. Due to the low recurrence and mortality rates associated with the surgical approach in LACC, the study is possibly underpowered to determine a significant difference in outcomes in our study. Given the very low number of complications, we were unable to adjust for complications during the follow up. We performed our analysis using standard hypothesis testing to identify the differences between the LC and OC approaches, which therefore could not confirm the comparability of these surgery approach. Thus, future studies using a formal equivalence or non-inferiority analysis are recommended to support this argument. Additionally, investigating prognostic differences between high-risk young and low-risk older CRC patients is an important area for future studies. Given the growing use of advanced techniques such as LC, further research into the potential benefits of LC specifically for younger patients would be timely. In summary, our results indicate that, there is no significant difference between LC approach and the open surgical approach in patients with LACC. However, a larger study may be necessary to detect the effects. Based on our estimates, a future study necessitates approximately a total of 324 patients to achieve 80% power for the detection of significant differences.

## Supporting information

S1 File OCvsLC_Deidentified_Dataset. (XLSX)
